# MCC950, the NLRP3 Inhibitor, Protects against Cartilage Degradation in a Mouse Model of Osteoarthritis

**DOI:** 10.1155/2021/4139048

**Published:** 2021-11-03

**Authors:** Bowei Ni, Wenbin Pei, Yunkun Qu, Rui Zhang, Xiangyu Chu, Yingguang Wang, Xiaojian Huang, Hongbo You

**Affiliations:** Department of Orthopedics, Tongji Hospital, Tongji Medical College, Huazhong University of Science and Technology, Wuhan, Hubei 430030, China

## Abstract

Osteoarthritis (OA), characterized by chronic systemic low-level inflammation and cartilage degeneration, is a type of arthritis closely associated with aging. Inflammation and aging play a pivotal role in the occurrence and progression of OA. NLRP3 inflammasome is involved in many inflammatory and aging diseases, and NLRP3 inhibitor MCC950 has anti-inflammatory and antisenescence effects on some diseases such as Alzheimer's disease. In the present study, we found that NLRP3 protein was upregulated in human and mouse OA cartilage. Moreover, NLRP3 and Caspase1 expression induced by IL-1*β* in chondrocytes was blocked by MCC950. In addition, MCC950 inhibited the expression of inflammatory mediators, matrix-degrading enzymes, senescence marker protein P16 (INK4A), and *β*-galactosidase, as well as excessive production of ROS. Meanwhile, MCC950 promoted autophagy-related protein expression and autophagy flux under the inflammatory condition. However, autophagy inhibitor 3-MA reversed anti-inflammatory and anticatabolic effects of MCC950. In in vivo experiments, intra-articular administration of MCC950 further showed its protective effect on cartilage degeneration. Bioinformatic analysis and in vitro experimental results revealed that MCC950 might play a protective role in cartilage by regulating Nrf2/HO-1/NQO1, PI3k/Akt/mTOR, P38/MAPK, and JNK/MAPK pathways. In conclusion, our work demonstrated that NLRP3 inhibitor MCC950 might serve as a promising strategy for OA treatment.

## 1. Introduction

Osteoarthritis (OA) affects more than 250 million people worldwide, among which middle-aged and elderly people are vulnerable [1]. OA is widely known as a disease of the whole joint, with the cartilage, synovial tissue, and subchondral bone to be regarded as the main lesions in the initiation and propagation of the disease [2]. Joint pain, swelling, and stiffness are common symptoms in OA, in which inflammation and aging play a crucial role [3, 4]. Current clinical practices mainly rely on nonsteroidal anti-inflammatory drugs to relieve symptoms such as pain, while the drug-related cardiovascular and gastrointestinal side effects cannot be avoided. Thus, there is an urgent need to explore new targeted drugs with fewer side effects.

Low-grade systemic chronic inflammation is one of the main characteristics of OA [5]. Inflammatory factors such as IL-1*β* can induce matrix degradation, oxidative stress, cell senescence, and impaired autophagy in chondrocytes, leading to cartilage degeneration [6]. Inflammasomes play a vital role in the initiation and development of inflammation via promoting the maturation and secretion of IL-1*β* [7]. NLRP3 (NOD-, LRR-, and pyrin domain-containing protein 3), which contributes to the occurrence of many inflammatory diseases such as rheumatoid arthritis (RA) [8], atherosclerosis [9], and gout [10], has received extensive attention in recent years. Moreover, numerous studies have shown that NLRP3 can interact with autophagy negative regulation protein mTOR and antioxidant protein Nrf2 to disrupt cell homeostasis [11, 12]. And inhibition of NLRP3 can promote autophagy [13] and alleviate oxidative stress [14] as well as aging-related diseases [15], indicating that NLRP3 might be a promising therapeutic target for OA.

MCC950, a selective inhibitor of NLRP3, inhibits the expression of Caspase1 and IL-1*β* [16], thereby blocking subsequent inflammation. In addition, MCC950 is qualified with the capacity of pain alleviation [17], antioxidation [18], autophagy regulation [19], and antiaging effect [20]. Moreover, MCC950 is supposed to enter the future clinical trial for Parkinson's disease owing to its antineuroinflammatory action [21]. Currently, the effect of MCC950 on synovitis in RA has been reported [22], whereas there are limited reports about the protective effect of MCC950 on chondrocytes.

The purpose of our study is to explore the probable effect and underlying mechanism of MCC950 on cartilage via in vivo and in vitro experiments, thus providing more clues for its therapeutic effect on OA.

## 2. Materials and Methods

### 2.1. Chemicals and Reagents

MCC950 was purchased from TOPSCIENCE. R&D Systems (501-RL-010, USA) provided mouse IL-1*β* cytokine. Primary antibodies against iNOS were obtained from Abcam (Shanghai, China). Components of the MAPK and PI3K/Akt/mTOR pathway, COX-2, Atg5/12, Beclin-1, and LC3A/B were acquired from CST (Beverly, MA, USA). In addition, Proteintech Group (Wuhan, Hubei, China) provided the corresponding primary antibody for GAPDH, MMP-13, collagen II, and components of the Nrf2/HO-1/NQO1 pathway. Primary antibody for ADAMTS5, secondary antibodies, collagenase type II, tyrisin, and phosphate buffer saline (PBS) buffer solution were purchased from Boster Biological Technology (Wuhan, Hubei, China). LC3 autophagy double-labeled adenovirus was acquired from Hanbio (Shanghai, China). The nuclear and cytoplasmic protein extraction kit, ROS assay kit, and senescence *β*-galactosidase staining kit were purchased from Beyotime (Shanghai, China).

### 2.2. Tissue Collection and Ethics Statement

Osteoarthritis human articular cartilage tissues were obtained from patients (*n* = 6) with knee OA undergoing total knee arthroplasty. The stage of OA was assessed by combining X-ray with the Kellgren-Lawrence grading scale. Normal human articular cartilage tissues were collected from patients (*n* = 6) without OA undergoing lower limb amputation owing to trauma. All patients were informed and signed consent. All experimental procedures were approved by the Ethical Committee of Tongji Medical College, Huazhong University of Science and Technology.

### 2.3. Identification of MCC950 Target Genes, Their Interaction Network, and OA-Related KEGG Pathways Containing MCC950 Target Genes

We predicted MCC950 target genes via using the PharmMapper database, then imported them into the Database for Annotation, Visualization, and Integrated Discovery (DAVID) for KEGG pathway enrichment analysis. To further explore the interaction of these genes, we used Cytoscape 3.7.2 and MCC algorithm to screen targeted genes and construct protein-protein interaction (PPI) networks. Moreover, we intersected KEGG pathways of these genes with OA-related pathways obtained from the miRWalk 2.0 database [23] to identify MCC950-related OA pathways. The Venn Diagram (Venny2.1, http://bioinfogp.cnb.csic.es/tools/venny/index.html) was used to show the common pathways.

### 2.4. Top KEGG Pathway Enrichment Analysis and Centrality Evaluation of MCC950 Target Genes

We selected the top six KEGG pathways from the intersection pathways and presented KEGG pathway enrichment analysis via using Pathway Builder Tool 2.0 (https://www.proteinlounge.com). Furthermore, we also evaluated the centrality of all the MCC950 target genes in the network by Cytoscape 3.7.2. Then, the degree, betweenness, and closeness centrality of MCC950 target genes in the network were presented in the string figure by using the circlize R package, which also included where genes are located on chromosomes and how they are connected.

### 2.5. Cell Viability Assay

Cytotoxicity of MCC950 on mouse articular chondrocytes was assessed by a CCK8 kit. Cells were seeded into 96-well plates in proper density (5000-10000/well). Then, stimulated with various concentrations (0.01, 0.1, and 1 *μ*M) of MCC950 for 24 h, 10 *μ*l CCK-8 solution was subsequently added to each well. Four hours later, a microplate reader (Bio-Rad, Richmond, CA, USA) at 450 nm was utilized to acquire OD (optical density) values of each well to assess the cell viability of chondrocytes.

### 2.6. Western Blotting

Each well in six-well plates was added into 100 *μ*l RIPA Lysis Buffer (1% of protease and phosphatase inhibitors) and laid on ice for 0.5 h. Then, the cell mixture was centrifugated at 12000 r/min for 0.5 h at 4°C. Protein concentration was evaluated by the OD value obtained by using a microplate reader (Bio-Rad, Richmond, CA, USA) at 562 nm. Nuclear proteins and cytoplasmic proteins were extracted by a kit (Beyotime, Shanghai, China) following the protocol. Denatured protein samples were subjected to 10% or 12% SDS-PAGE and transferred to PVDF membranes. After blocking with 5% skimmed milk for 1 h, the target proteins in the membrane were incubated with the correspondent primary antibodies for 16 h at 4°C, subsequently incubated with secondary antibodies for 1 h at room temperature. Supersensitive ECL chemiluminescent substrates (Yeasen, Shanghai, China) and an imager (Bio-Rad, USA) were utilized to develop the target proteins, and the related average optical density value of proteins was analyzed by ImageJ software.

### 2.7. mRFP-GFP-LC3 Adenovirus Infection

To observe the autophagy flux, mRFP-GFP-LC3 autophagy double-labeled adenovirus was used to overexpress LC3 protein and label red and green fluorescence. When chondrocyte convergence was about 50%, cells were infected with respective adenoviral vectors at a multiplicity of infection (MOI) of 20 as previously reported [24]. Owing to the acid sensitivity of green fluorescence, the green fluorescence will be quenched due to the formation of autolysosomes, and only red fluorescence will exist. Through counting the number of green and red nodes, respectively, the number of autophagosomes and intensity of autophagy could be assessed.

### 2.8. Quantification of ROS

The ROS assay kit (Beyotime, Shanghai, China) was utilized to determine and assess the production of ROS. After removing the medium containing serum, cells were incubated with the DCFH-DA fluorescence probe (10 *μ*M) prepared with serum-free medium for 30 min under a dark condition at 37°C, then detected by fluorescence microscopy and flow cytometry.

### 2.9. Senescence *β*-Galactosidase Staining

We used a senescence *β*-galactosidase staining kit to evaluate the activity of *β*-galactosidase. Cells were fixed for 15 min at room temperature, then incubated with staining fluid overnight at 37°C under no-CO_2_ condition, using light microscopy to observe and count the number of blue spots.

### 2.10. OA Mouse Model

Seven-week-old C57 male mice were subjected to three groups (*n* = 6 per group) at random, including the sham group, the DMM group, and the DMM+MCC950 group. After one week of adaption to the environment, C57 male mice were utilized for experiments. We just opened the joint cavity of mice anesthetized by intraperitoneal injection of pentobarbital (35 mg/kg) in the sham group on the right knee joint but further conducted destabilization of medial meniscus (DMM) surgery in the other two groups. After opening the joint cavity, the medial meniscus tibial ligament was cut off to destabilize the mobility of the medial meniscus. One week after the surgery, saline solution was injected into the joint cavity of mice in the sham and DMM groups, and mice in the DMM+MCC950 group received MCC950 (3 mg/kg) intra-articular injection. These animals were asphyxiated to death with CO_2_ after eight weeks of intra-articular injection. The right knee joint (upper tibia and lower femur) was fixed with 4% paraformaldehyde for 24 h, then decalcified in EDTA-NaOH solution on a horizontal shaker for 1 to 2 weeks. And the knee joints were dehydrated and embedded in paraffin. 6 mm sagittal sections were taken through the entire joint at 80 mm intervals. Slides were stained with Safranin-O and fast green. The degradation degree of mouse articular cartilage was evaluated according to the Osteoarthritis Research Society International (OARSI) score. Moreover, the expression of NLRP3, MMP13, Beclin-1, and Nrf2 in mouse knee articular cartilage was also evaluated by immunochemical staining.

### 2.11. Statistical Analysis

All experiments were repeated three times independently. The data was analyzed by GraphPad Prism v.7.01 software (GraphPad Inc., La Jolla, CA, USA). The results are shown as means ± S.D. Student's *t*-test was used to compare differences between any two groups. One-way analysis of variance (ANOVA) was utilized to determine differences among two or more groups, followed by a Tukey test. *P* value < 0.05 was considered significant.

## 3. Results

### 3.1. NLRP3 Expression Was Enhanced in OA Human Knee Articular Cartilage

**Figure 1:**
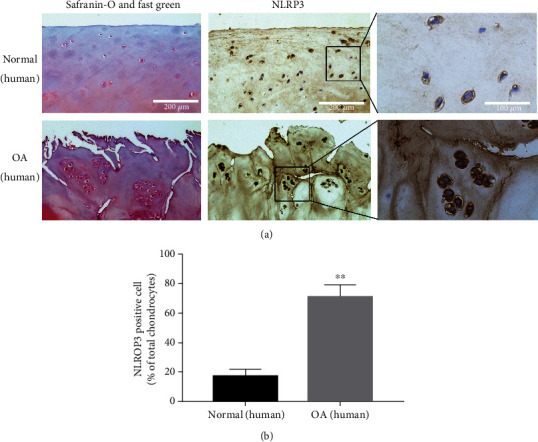
NLRP3 expression was enhanced in human OA cartilage. (a) Safranin-O and fast green and immunohistochemical staining of human knee articular cartilage. Scale bar: 200 *μ*m and 100 *μ*m. (b) Quantification analysis of NLRP3 expression in human cartilage. Data are shown as the mean ± SD. Significant differences between groups are indicated as ^∗^*P* < 0.05, ^∗∗^*P* < 0.01, and ^∗∗∗^*P* < 0.001 vs. normal group.

In our study, we detected the expression of NLRP3 in both normal and OA cartilage. Results (Figures 1(a) and 1(b)) showed that the number of NLRP3 positive cells in OA cartilage was much higher than in normal cartilage, especially in areas where the cartilage was worn away, which showed a potential association between NLRP3 and cartilage degeneration.

### 3.2. MCC950 Inhibited NLRP3 Expression in IL-1*β*-Treated Mouse Chondrocytes

**Figure 2:**
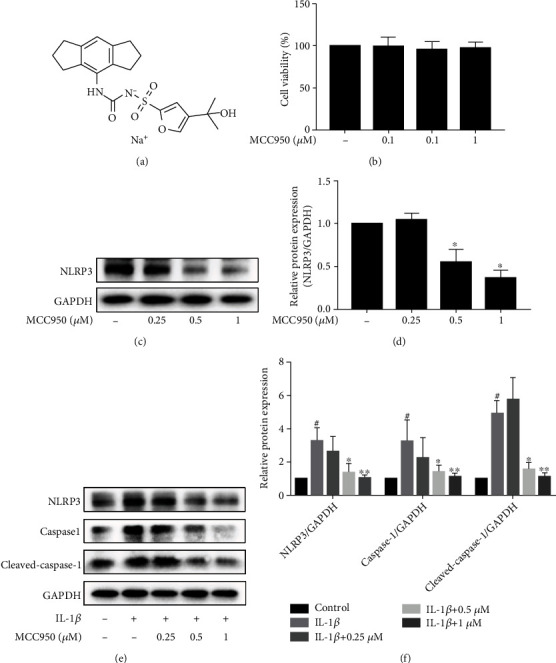
MCC950 inhibited NLRP3 expression in IL-1*β*-treated chondrocytes. Mouse chondrocytes were stimulated with 5 ng/ml IL-1*β* for 24 h after pretreatment with MCC950 for 2 h. (a) Chemical formula of MCC950. (b) CCK-8 assay of MCC950. (c) Western blotting results of NLRP3 expression in mouse chondrocytes only treated with MCC950 or not. (d) Quantification analysis of western blotting results. ^∗^*P* < 0.05 vs. control group. (e) Western blotting results of NLRP3, Caspase1, and Cleaved-Caspase1 expression in mouse chondrocytes treated with MCC950 with or without IL-1*β*. (f) Quantification analysis of western blotting results. Data are shown as the mean ± SD. Significant differences between groups are indicated as ^#^*P* < 0.05 vs. control group; ^∗^*P* < 0.05, ^∗∗^*P* < 0.01, and ^∗∗∗^*P* < 0.001 vs. IL-1*β* group.

We found that MCC950 was safe to chondrocytes at suitable concentrations ranging from 0.01 to 1 *μ*M through the CCK8 cytotoxicity assay (Figure 2(b)). MCC950 inhibited the expression of NLRP3 both in normal mouse chondrocytes (Figures 2(c) and 2(d)) and IL-1*β*-treated mouse chondrocytes (Figures 2(e) and 2(f)) in a dose-dependent manner. Meanwhile, Caspase1 and Cleaved-Caspase1, downstream molecule of NLRP3, were also inhibited at the protein level in inflammatory conditions.

### 3.3. MCC950 Repressed Inflammation and Catabolism in IL-1*β*-Treated Chondrocytes

**Figure 3:**
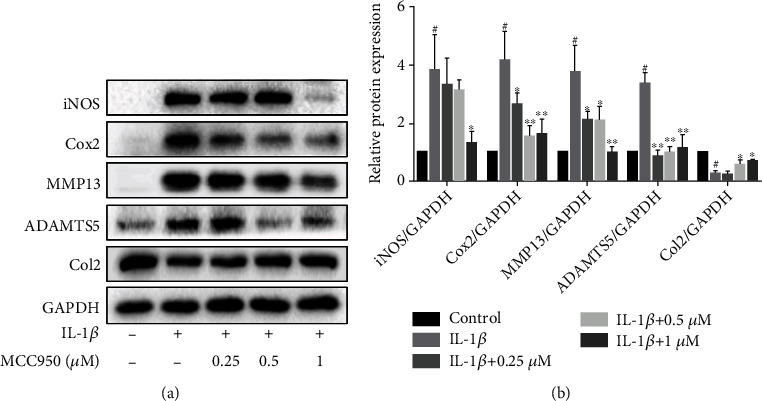
Anti-inflammatory and anti-catabolic effects of MCC950. Mouse chondrocytes were pretreated with MCC950 for 2 h, then stimulated with 5 ng/ml IL-1*β* for 24 h. (a) Western blotting results of inflammatory mediators and matrix-degrading enzymes. (b) Quantification analysis of western blotting results. Data are shown as the mean ± SD. Significant differences between groups are indicated as ^#^*P* < 0.05 vs. control group; ^∗^*P* < 0.05, ^∗∗^*P* < 0.01, and ^∗∗∗^*P* < 0.001 vs. IL-1*β* group.

To further evaluate the anti-inflammatory and catabolism regulatory effect of MCC950 on chondrocytes, we detected the expression of inflammatory, anti-catabolic, and anabolic mediators. Under the stimulation of IL-1*β*, the protein expression of iNOS, Cox2, MMP13, and ADAMTS5 was substantially upregulated, and Col2 expression was highly downregulated (Figures 3(a) and 3(b)). However, MCC950 significantly reversed the increase of inflammatory and anti-catabolic factor expression and rescued the decrease of Col2 expression.

### 3.4. MCC950 Ameliorated Autophagy Downregulation in IL-1*β*-Treated Chondrocytes

**Figure 4:**
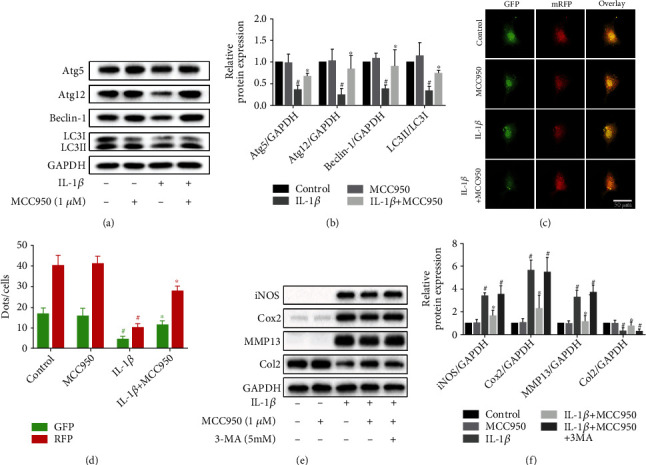
Regulation of autophagy by MCC950. (a, b) Western blotting results and quantification analysis of autophagy-related protein expression, including Atg5, Atg12, Beclin-1, and LC3I/II in chondrocytes treated with IL-1*β* with or without MCC950. ^#^*P* < 0.05 vs. control group; ^∗^*P* < 0.05 vs. IL-1*β* group. (c, d) Autophagy detection of chondrocytes transfected with the mRFP-GFP-LC3 adenovirus. Autophagosomes were represented by yellow puncta and autolysosomes by red puncta in merged images. ^#^*P* < 0.05 vs. control group; ^∗^*P* < 0.05 and ^∗∗^*P* < 0.01 vs. IL-1*β* group. Scale bar: 50 *μ*m. (e, f) Western blotting results and quantification analysis of inflammatory mediators and matrix-degrading enzymes in chondrocytes treated with or without 3-MA (5 mM) before treatment with MCC950 or IL-1*β*. ^#^*P* < 0.05 vs. control group; ^∗^*P* < 0.05 vs. IL-1*β* group.

Autophagy plays a critical role in the regulation of inflammation, anabolism, and catabolism. Thus, we aimed to identify whether the anti-inflammatory effect of MCC950 is associated with autophagy. It was observed that the expression of autophagy-related proteins, such as Atg5, Atg12, Beclin-1, and LC3II (Figures 4(a) and 4(b)), as well as the number of autophagosomes and autolysosomes (Figures 4(c) and 4(d)), was decreased under the stimulation of IL-1*β*. When pretreated with MCC950, the autophagy-related protein expression and the amount of autophagosomes were increased compared with the IL-1*β* group. Then, we found that 3-MA, an autophagy inhibitor, abolished the anti-inflammatory and anticatabolic effect of MCC950 (Figures 4(e) and 4(f)).

### 3.5. MCC950 Reduced IL-1*β*-Induced Oxidation Stress in Chondrocytes

**Figure 5:**
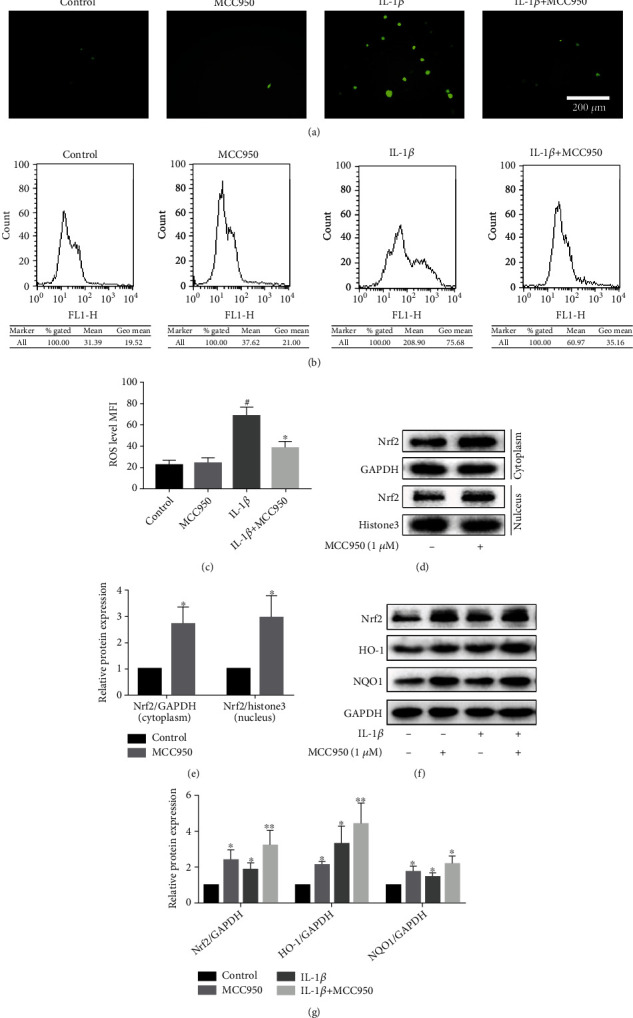
Antioxidative effect of MCC950. Mouse chondrocytes were pretreated with MCC950 for 2 h, then stimulated with 5 ng/ml IL-1*β* for 24 h. (a) ROS production observed under a fluorescence microscope. Scale bar: 200 *μ*m. (b) Flow cytometry results of ROS production. (c) Quantification analysis of the mean fluorescence intensity (MFI) of ROS in chondrocytes. ^#^*P* < 0.05 vs. control group; ^∗^*P* < 0.05 vs. IL-1*β* group. (d, e) Western blotting results and quantification analysis of Nrf2 nuclear/plasma protein expression. ^∗^*P* < 0.05 vs. control group. (f, g) Western blotting results and quantification analysis of Nrf2/HO-1/NQO1 pathway proteins. ^∗^*P* < 0.05 and ^∗∗^*P* < 0.01 vs. control group.

It is reported that IL-1*β* could promote the expression of ROS [25, 26], and excessive production of ROS is a potent inducer for OA. We therefore investigated the possible effect of MCC950 on the production of ROS and explored the underlying mechanism. We found that administration of MCC950 effectively suppressed the production of substantial ROS (Figures 5(a)–5(c)) incurred by IL-1*β* stimulation in chondrocytes both from the observation of fluorescent microscope and flow cytometry. Based on this, we further detected the expression of Nrf2/HO-1/NQO1 pathway protein under the stimulation of MCC950. As anticipated, nuclear protein and plasma protein expression of Nrf2 was enhanced significantly after being treated with MCC950 alone (Figures 5(d) and 5(e)). Consistent with the data reported previously [27], we found that IL-1*β* increased Nrf2 expression, and pretreatment with MCC950 could further enhance the expression of Nrf2, HO-1, and NQO1 proteins (Figures 5(f) and 5(g)).

### 3.6. Inhibitory Effect of MCC950 on Cell Senescence-Related Indicators in Chondrocytes

**Figure 6:**
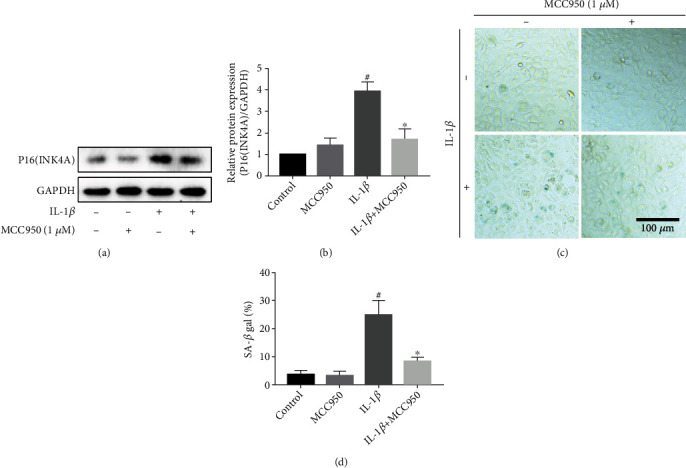
Inhibitory effect of MCC950 on senescence phenotype. Mouse chondrocytes were stimulated with 5 ng/ml IL-1*β* for 24 h after pretreatment with MCC950 for 2 h. (a, b) Western blotting results and quantification analysis of senescence marker protein P16 (INK4A). (c) *β*-Galactosidase staining of chondrocytes. Scale bar: 100 *μ*m. (d) Quantification analysis of *β*-galactosidase activity. Data are shown as the mean ± SD. Significant differences between groups are indicated as ^#^*P* < 0.05 vs. control group; ^∗^*P* < 0.05 and ^∗∗^*P* < 0.01 vs. IL-1*β* group.

Chondrocyte senescence is a typical phenomenon in the progression of OA, and inflammation is a paramount inducer of aging. Under the stimulation of IL-1*β*, senescence marker protein P16 (INK4A) expression was enhanced significantly. However, MCC950 reduced the production of P16 protein in IL-1*β*-treated chondrocyte without influencing its basal expression (Figures 6(a) and 6(b)). Moreover, we also assessed the activity of *β*-galactosidase which is an essential enzyme involved in regulating the aging process. In normal chondrocytes, the expression of *β*-galactosidase is low, but it has high activity in IL-1*β*-treated chondrocytes. Pretreatment with MCC950 could effectively inhibit this phenomenon (Figures 6(c) and 6(d)), which was correlated with the western blot result.

### 3.7. Bioinformatic Analysis of MCC950 Target Genes and OA-Related KEGG Pathways

**Figure 7:**
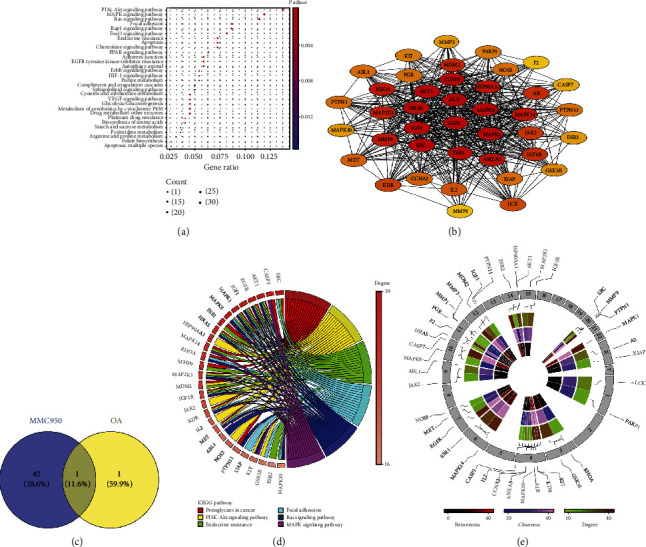
Bioinformatic analysis of MCC950 target genes and pathways. (a) KEGG pathway enrichment analysis of MCC950 target genes. (b) Interaction network diagram of MCC950 target genes. (c) Intersection of MCC950 KEGG pathways and OA-related pathways (Veen Diagram). (d) Top 6 pathways in intersection pathways (KEGG enrichment chord chart). (e) Centrality evaluation and chromosomal determination of hub genes of MCC950. Loci, connections, and name of the MCC950 target gene are shown on the outermost edge, and the lines that each gene connects represent the specific chromosome on which it is located.

We utilized bioinformatic methods to ascertain the potential target genes of MCC950 on the basis of the PharmMapper database. Through KEGG pathway enrichment analysis of these target genes (Figure 7(a)), we found that PI3K/Akt, MAPK, and Ras signaling pathways were the three pathways enriched with the most genes. To further clarify the association among MCC950 target genes, we selected 40 genes to construct the PPI network (Figure 7(b)). Among these genes, Akt1, MAPK8, SRC, ALB, and CASP3 are regarded as the top five genes according to the results of weight assessment. Then, 59 MCC950-related pathways were obtained through KEGG pathway enrichment analysis of the 40 genes. Meanwhile, we screened 105 OA-related pathways from the miRWalk2.0 database. On this basis, 17 common pathways (Figure 7(c)) were obtained by taking the intersection of the KEGG pathway of the 40 genes above and the OA-related pathway; thus, OA-related pathways that MCC950 may act on were acquired.

### 3.8. Top KEGG Pathways and Hub Genes of MCC950 Target Genes

Figure 7(d) shows the enrichment information of the top six KEGG pathways of MCC950 target genes, including proteoglycan in cancer, MAPK, PI3K/Akt, endocrine resistance, focal adhesion, and Ras pathways, and the six pathways were also included in OA-related pathways. We also found that SRC, CASP3, and Akt1 are the top three genes according to their own degree. Moreover, Akt1 was regarded as the hub gene owing to its association with all the top six pathways. The circular diagram (Figure 7(e)) represents the chromosomal position and connectivity of the MCC950 target genes. Among these genes, Akt1 also shows a greater degree, betweenness, and closeness centrality.

### 3.9. MCC950 Inhibited the Activation of PI3K/Akt/mTOR and MAPK Pathways in IL-1*β*-Treated Mouse Chondrocytes

**Figure 8:**
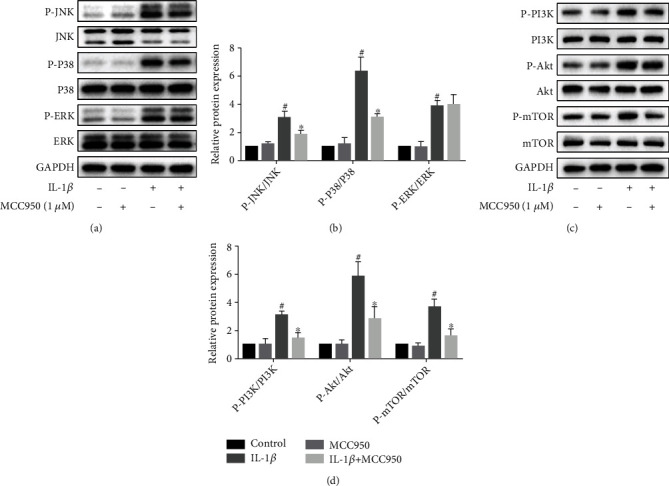
MCC950 inhibited activation of MAPK and PI3K/Akt/mTOR pathways in IL-1*β*-treated chondrocytes. (a, b) Western blotting results and quantification analysis of MAPK pathway proteins. (c, d) Western blotting results and quantification analysis of PI3K/Akt/mTOR pathway proteins. Data are shown as the mean ± SD. Significant differences between groups are indicated as ^#^*P* < 0.05 vs. control group; ^∗^*P* < 0.05 vs. IL-1*β* group.

PI3K/Akt/mTOR and MAPK pathways, which are closely linked to inflammation, metabolism, and autophagy, play a key role in the development of OA. Meanwhile, in order to verify the prediction results of bioinformatic analysis, we measured the protein expression of the two pathways. Results (Figures 8(a)–8(d)) showed that IL-1*β* significantly promoted the activation of these pathways, whereas pretreatment with MCC950 effectively blockaded their activation which manifests as phosphorylation. In addition to the ERK/MAPK pathway, phosphorylation of other pathway proteins was also repressed markedly by MCC950.

### 3.10. Protective Effect of MCC950 on Mouse Knee Articular Cartilage

**Figure 9:**
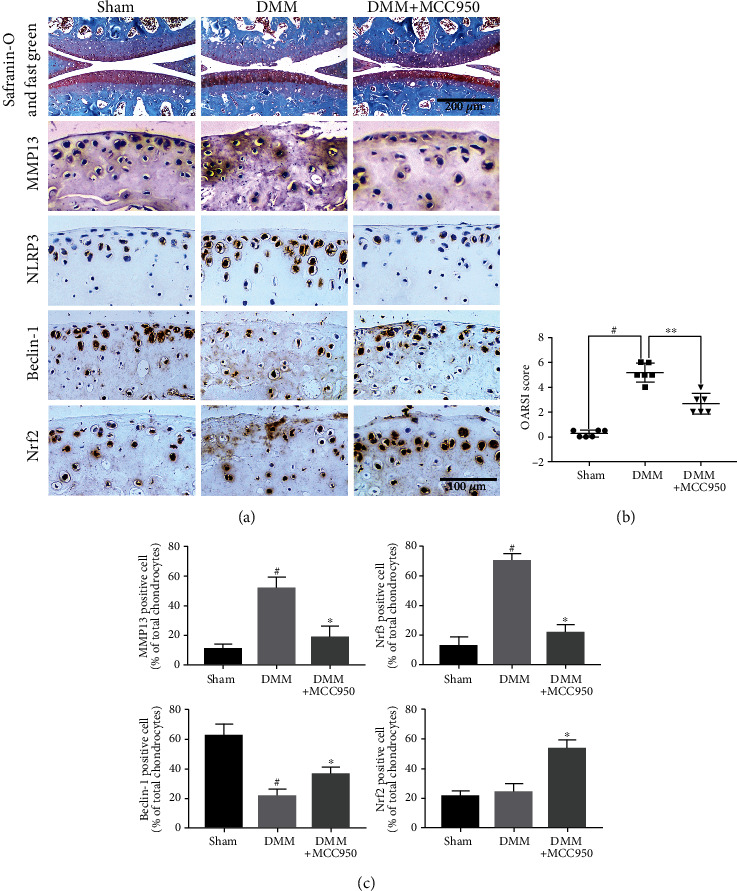
MCC950 ameliorated cartilage destruction in a mouse OA model in vivo. (a) Safranin-O and fast green and immunochemical staining of MMP13, NLRP3, Beclin-1, and Nrf2 expression. Scale bar: 200 *μ*m and 100 *μ*m. (b) Quantification analysis of OARSI score of knee cartilage in mice. (c) Quantification analysis of immunochemical staining of MMP13, NLRP3, Beclin-1, and Nrf2 expression. Data are shown as the mean ± SD. Significant differences between groups are indicated as ^#^*P* < 0.05 vs. sham group; ^∗^*P* < 0.05 vs. IL-1*β* group.

Degradation of cartilage is a typical characteristic of OA. In our experiment, we used the DMM model to simulate the process of OA and observed the wear and peeling of cartilage in the DMM group (Figures 9(a) and 9(b)) compared with the sham group. After MCC950 (3 mg/kg) was injected into the articular cavity for 8 weeks, amelioration of cartilage degradation could be observed. Moreover, the OARSI score in the DMM+MCC950 (3 mg/kg) group was lower than that in the DMM group. In addition, we also evaluated the expression of NLRP3, MMP13, Beclin-1, and Nrf2 in mouse cartilage via using immunochemical staining. Compared with the sham group, cartilage in the DMM group showed more NLRP3 positive and MMP13 positive chondrocytes, but less Beclin-1 positive chondrocytes. However, there were nonsignificant differences in the number of Nrf2 positive chondrocytes between the two groups (Figures 9(a) and 9(c)). In contrast to the DMM group, NLRP3 and MMP13 positive chondrocytes were decreased significantly, but Beclin-1 and Nrf2 positive chondrocytes were increased in the DMM+MCC950 (3 mg/kg) group.

## 4. Discussion

Inflammation plays an important role in the occurrence and development of OA. For example, substantial inflammatory factors were produced during the process of synovitis, thus contributing to the production of matrix-degrading enzymes and promoting cartilage degeneration consequently [28]. In addition, endogenous inflammation of chondrocytes also plays an important role [29]. NLRP3 inflammasome has been reported to be involved in hydroxyapatite-induced synovitis of the knee [30], and the application of NLRP3 inhibitor MCC950 can also significantly ameliorate RA [22]. Our study showed that MCC950 decreased the expression of NLRP3 in normal chondrocytes and IL-1*β* stimulated chondrocytes and reduced the wear of articular cartilage in OA mice. Therefore, we proposed that NLRP3 may be directly involved in the degeneration of cartilage in OA. MCC950 may play a protective role by inhibiting the expression of NLRP3 in articular cartilage and synovial membrane and thereby inhibiting the inflammatory response mediated by NLRP3.

Inflammatory mediators are paramount for the development of OA, among which IL-1*β* is of utmost importance to the activation of various inflammatory pathways, such as NF-*κ*B, MAPK, and PI3K/Akt pathways, leading to activation of downstream inflammatory proteins (iNOS, Cox2) and catabolism-related enzymes (MMP13, ADAMTS5) [31], which are involved in cartilage degradation. Multiple lines of evidence suggested that inhibition of these pathways can significantly reduce the expression of inflammatory factors and matrix-degrading enzymes and ameliorate cartilage degeneration [32, 33]. As an upstream activator of IL-1*β*, NLRP3 plays a key role in the maturity and secretion of IL-1*β*. Our results showed that MCC950 inhibited the activation of P38/JNK/MAPK and PI3K/Akt pathways, whereas MCC950 has no effect on the activation of the P65/NF-*κ*B pathway (Supplementary Figure 1). Meanwhile, iNOS, Cox2, MMP13, and ADAMTS5 were also downregulated, and Caspase1 protein directly related to the activation of IL-1*β* was also inhibited. Therefore, MCC950 may exert anti-inflammatory and anti-catabolic effects by inhibiting the activation of IL-1*β* and inflammation-related pathways.

Autophagy is an important physiological function of cells to clean up and reuse damaged organelles and macromolecules [34]. Late OA cartilage shows a decrease of the autophagy level, while activation and recovery of autophagy can play a protective role in OA [35, 36]. As a negative autophagy regulatory protein, mTOR is considered a promising therapeutic target for OA. Previous studies have shown that NLRP3 can interact with mTOR to promote the phosphorylation of mTOR [37], and mTOR can also participate in the ROS-induced activation of NLRP3 [11]. In our experiment, NLRP3 protein expression and mTOR phosphorylation were both increased in chondrocytes after stimulation with IL-1*β*, while MCC950 could not only downregulate NLRP3 expression but also suppress mTOR phosphorylation in IL-1*β*-treated chondrocytes. Meanwhile, the expression of autophagy-related proteins and the formation of autophagosome also recovered accordingly. These results indicated that under the IL-1*β*-induced inflammatory condition, NLRP3 may inhibit autophagy through interaction with mTOR, thus participating in the pathogenesis of OA, and MCC950 can protect against the detrimental change through inhibiting this pathological process. In addition, we found that the expression of inflammatory and catabolic mediators was increased after treatment with an autophagy inhibitor 3-MA, which weakened the protective effect of MCC950, suggesting that autophagy is involved in the protective effect of MCC950 on chondrocytes. However, further studies are merited to confirm whether the autophagy regulatory effect of MCC950 acts directly on the autophagy pathway or by interfering with the interaction between NLRP3 and mTOR.

Oxidative stress and aging are important causes of organ damage and degeneration. Excess production and accumulation of ROS can destroy the homeostasis of intracellular redox reaction, damage the mitochondrial function of chondrocytes, lead to senescence and death of chondrocytes, and eventually trigger cartilage degeneration [38]. Moreover, chronic inflammation is regarded as a major cause of chronic oxidative stress and aging [39]. Nrf2, as a key node in the regulation of oxidative stress, is involved in the regulation of ROS production, inflammation, and catabolism [27]. The experimental results showed that MCC950 can promote the entry of Nrf2 into the nucleus and activation of the Nrf2/HO-1/NQO1 pathway, inhibiting the excessive production of ROS. In addition, it could suppress the activity of *β*-galactosidase and the expression of P16 (INK4A) protein. The results suggested that MCC950 may exert a protective effect on cartilage by inhibiting oxidative stress and ameliorating senescence.

Cartilage degeneration is one of the characteristic lesions of OA, and delaying degeneration and promoting regeneration are the two intervention methods currently [40]. The results of animal experiments in this study showed that MCC950 can ameliorate the degeneration of OA cartilage, which was manifested by a lower OARSI score and ECM loss. Meanwhile, MCC950 markedly decreased NLRP3 and cartilage degrading enzyme MMP13 and upregulated the expression of autophagy markers like Beclin-1 and antioxidative translator Nrf2. These in vivo data further validated the protective effect of NLRP3 inhibitor MCC950 on OA.

In conclusion, it was found that the expression of the inflammasome protein NLRP3 was significantly upregulated in mouse OA cartilage, and the expression of inflammasome proteins was well inhibited by MCC950. Meanwhile, MCC950 also activated Nrf2/HO-O1/NQO1 and inhibited MAPK and PI3K/Akt/mTOR pathways to play antioxidant, anti-inflammatory, inhibition of catabolism, recovery of autophagy, and antisenescence effect to delay cartilage degeneration. This study provides a new idea for the cartilage protective effect of MCC950, suggesting that MCC950 may inhibit synovitis and also exert a direct cartilage protective effect via inhibiting the endogenous inflammation of cartilage. At present, the role of NLRP3 in the occurrence and development of OA still needs to be further studied, such as the role and mechanism of NLRP3 in the subchondral bone and other OA affected parts, to fully explain the pathogenesis of OA and explore new therapies.

## Data Availability

The data used to support the findings of this study are included within the article.

## Supplementary Materials

Supplementary Materials Supplementary Figure 1: MCC950 did not inhibit the activation of the P65/NF-*κ*B pathway in IL-1*β*-treated chondrocytes.
