# Supplementary material for: MCC950, the NLRP3 Inhibitor, Protects against Cartilage Degradation in a Mouse Model of Osteoarthritis
Source: Oxid Med Cell Longev. 2021 Nov 3;2021:4139048. doi: 10.1155/2021/4139048 (PMC8580635; doi:10.1155/2021/4139048)
Supplement: Supplementary Materials — Supplementary Figure 1: MCC950 did not inhibit the activation of the P65/NF-κB pathway in IL-1β-treated chondrocytes. [file 4139048.f1.pdf]

Supplementary Figure.1

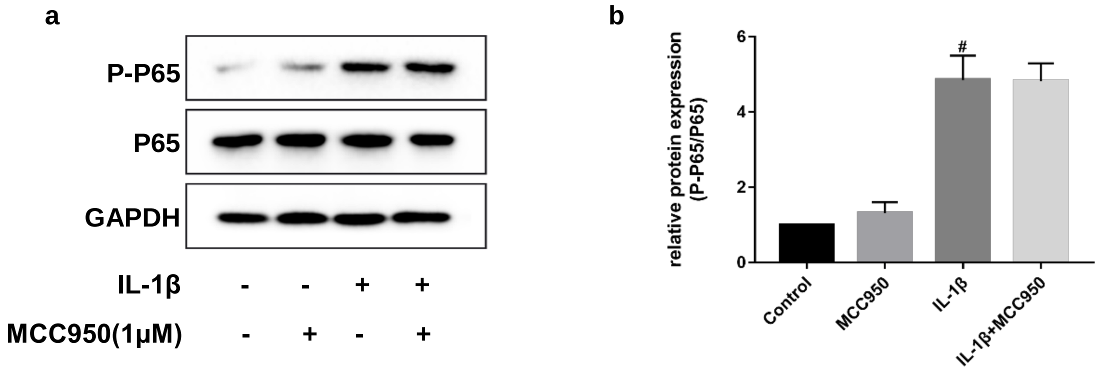

Supplementary Figure.1: MCC950 did not inhibit the activation of P65/NF-κB pathway in IL-1β-treated mouse chondrocytes. (a)Western blotting results of P65/NF-κB pathway proteins. (b) Quantification analysis of P65/NF-κB pathway proteins expression. Data are shown as the mean ± SD. Significant differences between groups are indicated as #P<0.05 vs control group; *P<0.05 vs IL-1β group.
